# Cadaver transplantation in Recent Era: Is Cadaveric Graft Survival Similar to Living Kidney Transplantation?

**Published:** 2011-11-01

**Authors:** N. Simforoosh, S. Gooran, A. Tabibi, A. Bassiri, M. R. Ghraati

**Affiliations:** 1 *Department of Urology and Renal Transplantation, Urology and Nephrology Research Center, Shahid Labbafi Nejad Hospital, Shahid Beheshti University of Medical Science, Tehran, Iran.*; 2 *Department of Urology and Renal Transplantation, Isfahan University of Medical Science, Isfahan, Iran.*

**Keywords:** Living donor, Cadaver donor, Kidney transplantation, Survival analysis

## Abstract

Background: Renal transplantation is the procedure of choice for most of patients with end-stage renal disease. The graft, however can be procured from either cadaver or living donors.

Objective: To compare graft and patient survival among patients who underwent kidney transplantation from cadaver donor vs. living donor.

Methods: From April 2002 to February 2010, we performed 138 cadaver kidney transplantations. We reviewed and compared one-year graft and patient survival with 138 living kidney transplantations.

Results: One-year graft and patient survivals in cadaveric groups were 93% and 96%, respectively, and in living groups were 92% and 97%, respectively.

Conclusion: There was no significant difference in one-year graft and patient survival between living and cadaver donor kidney transplantation.

## INTRODUCTION

There is increasing number of patients with end-stage renal disease (ESRD), and renal transplantation is the procedure of choice for most of them, since it improves the quality of life and is cost-effective compared with dialysis [1-3].

The short-term outcome of renal transplantation has been improved substantially in the past 15 years [1]. Survival after renal transplantation is the most important outcome measure when transplantation results are analyzed.

Generally, it has been assumed that living donor kidney transplantation (LDKT) grafts are superior to deceased donor kidney transplantation (DDKT) grafts in terms of graft survival and a lesser recipients’ morbidity. Moreover, relative insufficient supply of cadaver kidneys can be compensated with living donors. Furthermore, specific planning for the operation, say pre-emptive transplantation, or limitation of the time on dialysis are all possible during living donor program [3, 4].

Since 1985, we have performed 3512 kidney transplantations in our department. The majority of these transplantations were living unrelated. We have previously found that the result of living related (non-HLA identical) and unrelated kidney transplantation are similar [5]. This study was conducted to compare one-year graft and patient survival among patients who underwent kidney transplantations from cadaver donors *vs*. living donors.

## PATIENTS AND METHODS

In this retrospective study, 138 patients who had received renal grafts from deceased donors were surveyed from April 2002 to February 2010. One-year graft and patient survival were compared to 138 patients who had received the grafts from living donors.

In living kidney transplantation group, all donors underwent laparoscopic donor nephrectomy (LDN). Live donors underwent extensive medical evaluation and were accepted for donation only if they were in good health. Those with hypertension, diabetes and proteinouria—even very mild—were not accepted for kidney donation. Donors underwent computerized or rarely conventional angiography for evaluation of renal vascular anatomy and those with multiple vessels were also excluded except if they were related with the recipient.

To increase the cost-effective of LDN, pure LDN was done without using endovascular staplers. Instead hemolock and titanium clips were used to ligate the renal artery and vein separately [6]. The first trocar was introduced in an open technique using an ordinary non-disposable trocar. The harvested kidney was extracted manually via an 8-10 cm suprapubic incision. The kidneys were transplanted by the same urologist who harvested the kidneys [6].

All cadaver kidneys were harvested in the same city but in another hospital specialized for cadaver surgery. Harvesting surgery and the recipient operation were coordinated in a way that the harvested kidney could be transferred to the recipient’s operating room in less than two hours with cold ischemia time less than three hours. Two pediatric cadavers less than six years were accepted and two kidneys were transplanted en bloc to adult recipients successfully.

Immunosuppression protocols were similar for the two groups, namely cyclosporine, azathioprine and prednisolone. Antithymocyte globulin (ATG) was used if needed.

Graft failure was defined as the need for renal function replacement therapy from any cause. Delay graft function (DGF) was defined as a serum creatinine level >3.5 mg/dL on the third day post-transplantation.

Patient and graft survival rates were analyzed by the Kaplan-Meier survival analysis for differences in patient and graft survivals as assessed by the Log-Rank test. A p value <0.05 was considered statistically significant.

## RESULTS

The studied patients included 276 kidney transplant patients (138 from cadaver donors and 138 from living donors). Demographic data of patients are shown in Table 1. Of 138 donors in living group, 132 (95.7%) were unrelated and six were related. Two patients experienced ureteral fistula and two experienced acute rejection.

**Table 1:** Demographic data of recipients from cadaveric *vs.* living donors

Characteristics	Cadaver donor (n=138)	Living donor (n=138)	p value
Gender, n(%)			0.9
Male	84 (60.8)	87 (63.1)	
Female	54 (39.2)	51 (36.9)	
Mean recipient age (yrs)	43.6	41.3	0.12
Mean donor age (yrs)	28.9	28.2	0.56

In cadaveric group, number of patients who had ureteral fistula and acute rejection were one and two, respectively. Three patients (2%) suffered from DGF in living group and five (3%) in cadaveric group. There were no conversions to open nephrectomy or reoperation in any of LDNs. 

In the living group, the mean duration of cold ischemia was 42 (range: 32–55) min and warm ischemia was 8.6 (range: 5–17) min. In cadaveric group, the mean duration of cold ischemia was less than three hours and warm ischemia was almost zero.

The results of one-year graft and patient survival were similar in cadaveric and living group. One-year graft and patient survivals in cadaveric groups were 93% and 96%, respectively, and in living groups were 92% and 97%, respectively. Using the Log-Rank test, these differences were not statistically significant (p=0.81 and p=0.78, respectively).

## DISCUSSION

In this study, we analyzed the outcome of 138 patients who had received cadaver renal grafts and compared with that of 138 patients who had received living renal grafts. The results of this study demonstrated that one year after transplantation, the patient and graft survivals were similar in cadaveric *vs*. living groups.

In 2005, we reported on the first randomized clinical trial comparing 100 LDNs with a similar number of open donor nephrectomies (ODNs) and concluded that compared to ODN, LDN was associated with a greater donor satisfaction, less donor morbidity and similar graft outcome [6].

Our results differed from other studies, which reported that living kidney transplantation had a better outcome than cadaveric transplantations. Mehrabi, *et al*, [7] reported that living related and unrelated kidney transplantations had better results than cadaveric transplantations.

Lee, *et al*, [4] reported that analysis of overall survival of kidney grafts showed significantly better survival in the living donor kidney transplantation (LDKT) group compared with the deceased donor kidney transplantation (DDKT) group. However, when the analysis was restricted to grafts surviving >5 years, the difference in survival was clearly attenuated; both groups showed similar rates of graft loss. From 1988 to 1996, one-year graft survival increased from 89% to 94% for recipients of living donors and from 77% to 88% for cadaveric donor recipients [1]. Hariharan, *et al*, [1] reported that the projected half-life of grafts has improved progressively from 7.9 to 13.8 years for a cadaveric donor and from 12.7 to 21.6 years for a living donor for the period from 1988 to 1995. This improvement was not totally attributable to any of the newer immunosuppressive drugs, as it took place in the era of treatment with cyclosporine, azathioprine, and prednisone. Given these findings, more attention has been paid not only to immunologic but also to non-immunologic strategies that might improve long-term outcomes through prevention of late allograft loss [8].

We think that one of the reasons for obtaining similar results in our study was the short period between harvesting and transplantation (<3 hrs) that was near to living transplantation. In contrast to popular belief [1, 3, 4, 7, 8], in our study, there was no significant difference in one-year graft and patient survival between living and cadaver donor kidney transplantation.

We therefore concluded that with improved harvesting techniques and early grafting of the harvested kidneys, graft and patient survival similar to living kidney transplantation could be obtained in cadaver transplantation.
